# Retraction Note: Overexpressed transient receptor potential vanilloid 1 (TRPV1) in lung adenocarcinoma harbours a new opportunity for therapeutic targeting

**DOI:** 10.1038/s41417-023-00677-0

**Published:** 2023-10-13

**Authors:** Yichu Nie, Fenglan Feng, Wei Luo, Andrew J. Sanders, Yidi Zhang, Jiaming Liang, Cheng Chen, Weineng Feng, Weiquan Gu, Weiping Liao, Wei Wang, Jinfeng Chen, Lijian Zhang, Wen G. Jiang, Jin Li

**Affiliations:** 1 Clinical Research Institute, The First People’s Hospital of Foshan & Sun Yat-sen University Foshan Hospital, Foshan, 528000 PR China; 2 School of Pharmaceutical Sciences (Shenzhen), Sun Yat-sen University, Guangzhou, 510275 PR China; 3 State Key Laboratory of Respiratory Diseases, the First Affiliated Hospital of Guangzhou Medical University, Guangzhou Medical University, Guangzhou, 510120 PR China; 4 CCMRC, Cardiff University School of Medicine, Cardiff, UK; 5 Foshan Fourth People’s Hospital, Foshan, 528000 PR China; 6 Peking University Cancer Hospital and Beijing Cancer Institute, Department of Thoracic Surgery, Fucheng Road, Haidian District, Beijing, China

**Keywords:** Non-small-cell lung cancer, Biomarkers

Retraction to: *Cancer Gene Therapy* 10.1038/s41417-022-00459-0, published online 30 March 2022

The Editor-in-Chief has retracted this article. After publication irregularities were noted in multiple figures, specifically:

In Figure 2E there is overlap between the images in the 2% CSE for A549 and HCI-N292.

Figure 4F the Rac1 band overlaps with the CREB band in figure 5C.

Furthermore, the authors were unable to provide the raw images of the blots published in this study. the The Editor-in-Chief has therefore lost confidence in the results presented in this article.

Yichu Nie does not agree to this retraction. Fenglan Feng, Wei Luo, Andrew J. Sanders, Yidi Zhang, Jiaming Liang, Cheng Chen, Weineng Feng, Weiquan Gu, Weiping Liao, Wei Wang, Jinfeng Chen, Lijian Zhang, Wen G. Jiang and Jin Li have not responded to any correspondence from the editor about this retraction.

